# Dietary Patterns in a Nationwide Cohort of Patients with Hereditary Fructose Intolerance

**DOI:** 10.3390/nu18050771

**Published:** 2026-02-27

**Authors:** Elsa Izquierdo-García, Edorta Mora, Dolores García-Arenas, Dámaris Martínez Chicano, María Soledad López-García, Carlos Alcalde, Amaya Belanger-Quintana, Elvira Cañedo-Villarroya, Leticia Ceberio, Estrella Diego, Marcello Bellusci, Silvia Chumillas-Calzada, Patricia Correcher, María-Luz Couce, Ainara Cano, Igor Gómez, Tomás Hernández, Montserrat Morales, Consuelo Pedrón-Giner, Estrella Petrina Jáuregui, Luis Peña-Quintana, Paula Sánchez-Pintos, Juliana Serrano-Nieto, María Unceta Suarez, Arantza Arza, Isidro Vitoria Miñana, Teresa C. Delgado, Javier de las Heras

**Affiliations:** 1 Pharmacy Department, Infanta Leonor University Hospital, 28031 Madrid, Spain; 2 Department of Pediatrics, University of the Basque Country (EHU), 48940 Leioa, Spain; edortamora@gmail.com; 3 Division of Pediatric Metabolism, CIBERER, Cruces University Hospital, 48093 Barakaldo, Spain; 4 Department of Pediatric Gastroenterology, Hepatology and Nutrition, Sant Joan de Déu Hospital, 08950 Barcelona, Spain; 5 Biobizkaia Health Research Institute, 48093 Barakaldo, Spainmaria.uncetasuarez@osakidetza.eus (M.U.S.);; 6 Pediatrics Unit, Río Hortega University Hospital, 47012 Valladolid, Spain; 7 Metabolic Diseases Unit, Department of Pediatrics, Ramon y Cajal Hospital, 28034 Madrid, Spain; 8 Department of Metabolism Diseases and Nutrition, Niño Jesús University Children’s Hospital, 28009 Madrid, Spaincpedronginer@gmail.com (C.P.-G.); 9 Internal Medicine Service, Cruces University Hospital, 48903 Barakaldo, Spain; 10 Endocrinology & Nutrition Department, Cruces University Hospital, 48093 Barakaldo, Spain; 11 12 de Octubre University Hospital, CIBERER, 28041 Madrid, Spain; 12 Nutrition and Metabolic Diseases Unit, La Fe University Hospital, 46026 Valencia, Spain; correcher_pat@gva.es (P.C.);; 13 Unit of Diagnosis and Treatment of Congenital Metabolic Diseases, Department of Pediatrics, IDIS-Health Research Institute of Santiago de Compostela, CIBERER, MetabERN, Santiago de Compostela University Clinical Hospital, 15704 Santiago de Compostela, Spain; 14 Araba University Hospital, 01009 Vitoria-Gasteiz, Spain; 15 Pediatric Service, Albacete University Hospital, 02006 Castilla-La Mancha, Spain; 16 Clinical Nutrition Section, Navarra University Hospital, 31008 Pamplona, Spain; 17 Pediatric Gastroenterology, Hepatology and Nutrition Unit, Mother and Child Insular University Hospital Complex, Asociación Canaria para la Investigación Pediátrica (ACIP), CIBEROBN, University Institute for Research in Biomedical and Health Sciences, University of Las Palmas de Gran Canaria, 35016 Las Palmas de Gran Canaria, Spain; 18 Pediatric Service, Málaga Regional University Hospital (HRU), 29010 Málaga, Spain; 19 Biochemistry Laboratory, Metabolism Area, Cruces University Hospital, 48903 Barakaldo, Spain; 20 IKERBASQUE, Basque Foundation for Science, 48009 Bilbao, Spain

**Keywords:** hereditary fructose intolerance, diet, fructose

## Abstract

**Background/Objectives**: Hereditary fructose intolerance (HFI) is an inherited metabolic disorder caused by a deficiency of the enzyme fructose-1,6-bisphosphate aldolase. Treatment consists of a lifelong diet restricted in fructose, sucrose, and sorbitol (FSS). The aim of this study was to determine dietary intake of FSS and to analyze the consumption patterns of vegetables, fruit, legumes, pulses, and dried fruit in a nationwide cohort of HFI patients. **Methods**: Overall, 36 HFI patients and 28 age-, sex- and BMI-matched healthy control subjects participated in this study. A self-administered three-day dietary record and an adapted quantitative food frequency questionnaire (FFQ) including frequency and portion sizes were collected. FSS intake was calculated using the DIAL Nutritional Calculation Program (ALCE INGENIERÍA). Total fructose intake was calculated as the sum of free fructose, 50% of sucrose, and sorbitol. **Results**: Protein intake was significantly higher in HFI patients compared to the controls (92.43 g/day [65.1–165.03] vs. 70.39 g/day [35.21–133.83]; *p* = 0.001). In most patients, total fructose intake was within the recommended limits (9.79 mg/kg bw/day [0.29–59.09]), with no significant differences between children and adults (*p* = 0.325). Although the established dietary recommendations did not always match the actual intake observed in a real-life setting, in general, foods with higher fructose content were consumed less frequently and in smaller quantities. **Conclusions**: Further research on the fructose content of various foods, particularly fruits and vegetables, and updated dietary recommendations for HFI patients are warranted to provide the best tools for the nutritional management of the disease.

## 1. Introduction

Hereditary fructose intolerance (HFI; OMIM 229600) is a rare autosomal-recessive inborn error of metabolism due to a deficiency of the enzyme fructose-1,6-bisphosphate aldolase (aldolase B; E.C. 4.1.2.13), with a prevalence of 1/10,000–1/20,000 [1,2]. Aldolase B is an important enzyme in fructose metabolism or fructolysis, allowing the breakdown of fructose 1-phosphate into metabolites of glycolysis, mainly in the liver but also in the renal tubules and the small intestine. Some of the metabolic disturbances that occur after fructose ingestion are due to the accumulation of fructose 1-phosphate, the product immediately upstream of the deficient enzyme. In the case of consumption of large amounts of fructose (4–6 g/kg body weight (bw)/day), rapid onset of symptoms occurs with nausea, vomiting, sweating, lethargy, shock, dehydration, hepatic and renal dysfunction and/or hypoglycemia (by block of glycogenolysis and gluconeogenesis by fructose 1-phosphate), which can lead to coma and even death. Moreover, with ongoing consumption of even small amounts of fructose (up to 250 mg/kg bw/day), HFI patients present feeding difficulties, vomiting, hepatomegaly and fatty liver [3], edema, ascites and failure to thrive [4,5], as well as other analytical alterations (hyperuricemia, hyperuricosuria, and increased plasma concentration and urinary excretion of magnesium) [1,6].

Usually, the first symptoms occur at around 6 months of life, when fructose-containing foods are introduced into the diet. Most patients are diagnosed less than 3 years after the onset of the first symptoms, or even earlier in recent years [7]. However, some cases of acute liver or multi-organ failure continue to be reported in undiagnosed HFI neonates and young infants as a result of the consumption of common infant formula containing sucrose [8]. In contrast, in some cases the diagnosis is delayed into adulthood due to HFI patients’ typical aversion to sweet foods, avoiding acute intoxication but not chronic intoxication or long-term complications.

Strong aversion to sweets is a typical feature of patients with HFI, as patients unconsciously modify their eating habits with respect to fruit, vegetables and sweets. Therefore, some patients at diagnosis, especially if they are not forced in childhood to eat certain foods that they reject, do not need to modify their diet substantially because they already strictly follow the dietary recommendations instinctively [9,10].

Fructose is naturally present in fruit and vegetables and also in sweeteners such as sucrose (fructose and glucose disaccharide) and honey. Fructose is also a component of other carbohydrates, such as raffinose, stachyose or polysaccharides such as inulin or fructooligosaccharides (FOSs), none of which are metabolized by human gastrointestinal enzymes. Sorbitol (which is converted to fructose by sorbitol dehydrogenase), polyols that contain sorbitol in their structure (maltitol, lactitol or isomaltitol), and sweeteners that are metabolized by the same metabolic pathway as fructose (tagatose) are not recommended in HFI patients [11].

Although there are some pharmacological treatments under investigation [12,13], currently the only treatment available for HFI patients is a fructose, sucrose, and sorbitol (FSS)-restricted diet for life (less than 40 mg/kg body weight (bw)/day of total fructose) [4]. However, in practice this FSS-restricted diet is difficult to follow because sucrose and sorbitol are used as ingredients in many processed foods; there is also variation in the amount of fructose in some vegetables, legumes or fruit, and the exact amount of fructose in many of them is unknown.

On the one hand, to evaluate the daily intake of fructose and other nutrients, a three-day food diary is a reliable indicator of a person’s eating habits. Due to variability in intake throughout the week, participants are usually requested to record data over two days during the week and one day on the weekend. Detailed information about food preparation methods, ingredients of dishes and recipes, and even the brand name of commercial products is often requested [14].

On the other hand, food frequency questionnaires (FFQs) are designed to collect information in a standardized format about the types and frequencies of foods consumed over a predetermined set of time. In quantitative FFQs, participants also estimate the portion size consumed either in household measures or grams. This method was designed to provide descriptive qualitative information about food-consumption patterns, and, depending on the interests of the researchers, FFQs may focus on the intake of specific foods or nutrients [15].

The aim of this study was to determine the dietary intake of fructose, sucrose and sorbitol, as well as the food-consumption patterns, frequency and servings of principal vegetables, fruit, legumes, pulses and dried fruit, in a nationwide cohort of Spanish HFI patients.

## 2. Patients and Methods

### 2.1. Study Participants

Study visits were carried out at Cruces University Hospital, Spain, from October 2019 to November 2020. The study population comprised 36 genetically diagnosed HFI patients and 28 healthy control subjects, who were selected to be matched for sex, age (±2 years), and BMI (±2 kg/m^2^). All HFI patients had been on an FSS-restricted diet for at least two years.

Twelve Spanish hospitals participated in the study: Cruces University Hospital, Basque Country [host]; Araba University Hospital, Basque Country; Navarra University Hospital, Navarra; 12 de Octubre University Hospital, Madrid; Niño Jesús University Children’s Hospital, Madrid; Ramón y Cajal University Hospital, Madrid; La Fe University Hospital, Valencian Community; Málaga Regional University Hospital, Andalusia; Santiago de Compostela University Clinical Hospital, Galicia; Río Hortega University Hospital, Castile and Leon; and Mother and Child Insular University Hospital complex, Canary Islands.

### 2.2. Ethics Approval and Consent to Participate

The study protocol was performed according to the ethical guidelines of the revised 1975 Declaration of Helsinki [16] and approved by the Research Ethics Committee of the Basque Country (CEIm-E), with ethic approval code PI2019072. Written informed consents were obtained from parents or legal guardians of children (below 18 years of age) and adult study participants.

### 2.3. Assessment of Diet

Dietary information was collected in a self-administered nutritional record of dietary intake over three days (two during the week and one on the weekend). The fructose, sucrose and sorbitol intake was calculated using the Nutritional Calculation DIAL Program (Version 3.10.5.0) of ALCEINGENIERIA (Bogotá, Colombia) [17]. When specific values were not available in the Nutritional Calculation DIAL Program (Version 3.10.5.0), the missing data were completed by calculating the mean of the values reported in other databases such as the Australian Food Composition Database—Release 1.0 [18], Danish Frida Food Database [19], Finnish food composition database [20] and German Food Composition and Nutrition Tables [21], which were also consulted to complete and provide more information. Total fructose consumption was calculated as the sum of free fructose, 50% of sucrose and sorbitol.

Furthermore, an adapted quantitative FFQ was carried out regarding 72 food items, including vegetables, fruit, pulses and legumes, and the frequency of consumption (times daily, weekly, monthly or yearly) and portion size (formerly called servings) in grams or household measures during the last year [15,22,23]. Manufactured products were excluded from the FFQ because their consumption is limited among patients with HFI.

### 2.4. Statistical Analysis

Data are presented as the mean ± standard deviation or median and range (minimum–maximum) depending on the data distribution. Continuous variables were compared using unpaired Student’s *t*-test or the Mann Whitney U test depending on the data distribution. Differences in categorical variables were assessed using the χ2 or Fisher’s exact test depending on the data distribution. *p*-values were based on two-tailed comparisons, and those less than 0.05 were considered to indicate a statistically significant difference. In order to assess bivariate relationships between variables, Pearson or Spearman Rank test correlation analyses were used. Statistical analyses were performed using SPSS software, version 20 for Windows (IBM, Chicago, IL, USA).

## 3. Results

### 3.1. Study Cohort and Dietary Intake

The study population comprised 36 genetically diagnosed HFI patients, all of them of Caucasian origin. The HFI patients were 16 males and 20 females, and 21 were under 18 years of age. There were no significant differences in sex, age, weight or BMI between HFI and healthy control subjects. Regarding the dietary intake of macronutrients, the consumption of protein was statistically higher in HFI patients compared to the control group (Table 1).

Total fructose intake (fructose, 50% of sucrose and sorbitol) was lower in HFI patients than in healthy controls (0.41 g/day [0.01–2.5] vs. 22.93 g/day [5.8–50.31]; *p* < 0.001) (Table 1). Only one HFI adult patient consumed more than 2 g/day of total fructose, and three patients consumed 1.5–2 g/day: two children (1.54 and 1.61 g/day) and one adult (1.57 g/day).

No statistically significant differences between children and adults were found in the dietary intake of fructose, sucrose and/or sorbitol per kg bw/day among HFI patients (Table 2). All HFI patients consumed a total amount of fructose below 59.09 mg/kg bw/day (Table 2). There were three HFI patients who consumed more than 40 mg/kg bw/day: one adult who consumed 46.2 mg/kg bw/day and two children with intakes of 52.8 and 59.09 mg/kg bw/day.

### 3.2. FFQ Results: Food Item Consumption

Table 3 and Table 4 show the results of the FFQ relating to “*Vegetable intake*” and “*Fruit*, *dried fruit*, *pulse and legume intake*” in HFI patients.

Among HFI patients, over the last year, old potato (97.2%), spinach (88.9%), mushrooms (86.1%) and chard (77.8%) were the vegetables that were consumed most; there were no statistically significant differences in their consumption between children and adults (Table 3). However, other less-consumed vegetables such as cauliflower, asparagus, some kinds of lettuce, arugula and carrot were consumed more frequently by adult HFI patients (Table 3). Only one patient indicated that they consumed a vegetable that was not on the questionnaire list, in this case borage.

Regarding consumption of fruit, only olives (green and black olives), avocado and lemon were consumed by most HFI patients (77.8%, 52.8%, 61.1% and 58.3% respectively). Nuts and dried fruit were consumed, mainly sunflower seeds (75%), and some pulses and legumes such as lentils and chickpeas (80.6% and 72.2% respectively) (Table 4).

### 3.3. FFQ Results: Consumption Frequency

**Figure 1:**
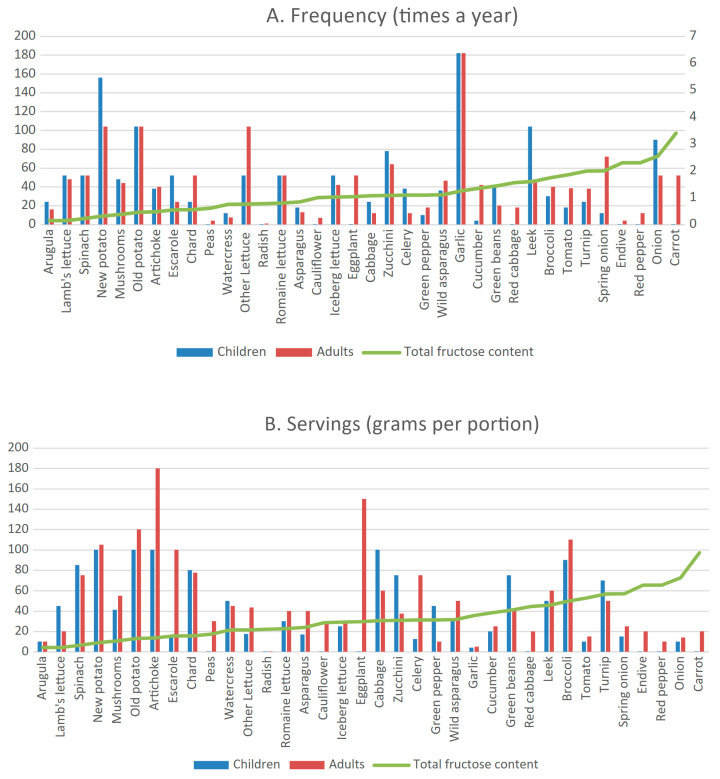
Frequency (**A**) and portion sizes in grams per portion (**B**) of vegetable intake by HFI patients. Data are represented as medians due to data distribution. Secondary axis and solid line represent amount of total fructose (fructose + 50% sucrose + sorbitol) in each food per 100 g edible portion.

Garlic (182 times a year [12–1278]), new and old potato (104 times a year [8–730] and 104 times a year [2–365]), zucchini (78 times a year [2.5–312]) and leek (72 times a year [3–312]) were the most frequently consumed vegetables among HFI patients who consumed these foods (Figure 1A). There were no statistically significant differences in consumption frequency between children and adults for any of the vegetables analyzed. 

**Figure 2:**
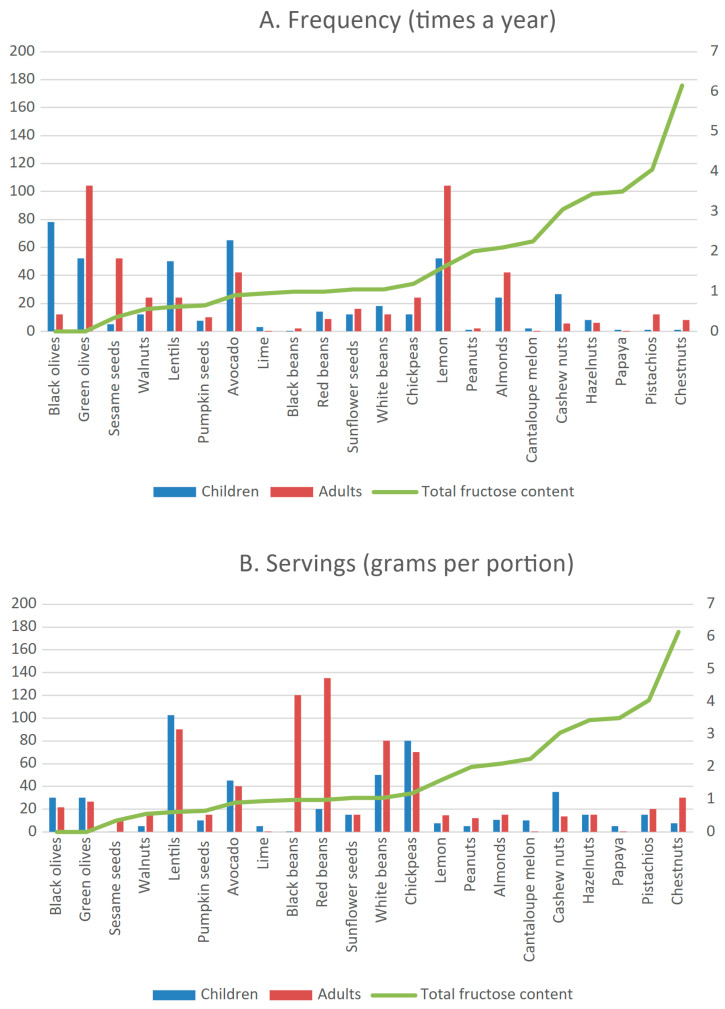
Frequency (**A**) and portion sizes in grams per portion (**B**) of fruit, dried fruit, pulse and legume intake by HFI patients. Data are represented as medians due to data distribution. Secondary axis and solid line represent amount of total fructose (fructose + 50% sucrose + sorbitol) in each food per 100 g edible portion.

Green olives (78 times a year [3–365]) and lemon (104 times a year [2–364]) were frequently consumed. Lentils were the most widely consumed pulses (48 times a year [12–156]). Pistachios (3.5 times a year [1–20]) and chickpeas (24 times a year [4–104)] were not often consumed, but they were more frequently consumed by adult than pediatric HFI patients (pistachios = children: 1 time a year [1–2] vs. adults: 12 times a year [5–20]; *p* = 0.08) and (chickpeas = children: 12 times a year [4–52] vs. adults: 24 times a year [15–104]; *p* = 0.05) (Figure 2A).

The median frequency of total vegetable and fruit consumption per day was 4.94 times a day [2.47–8.82] in healthy controls and 2.87 times daily in HFI patients [0.08–6.90] (*p* < 0.001). Regarding legume intake, there was a statistical trend for lower weekly consumption in HFI patients compared to the healthy control subjects (2.04 times weekly [0.65–7] in healthy controls and 1.17 times weekly [0.87–4.38] in HFI patients; *p* = 0.054).

### 3.4. FFQ Results: Portion Sizes

The servings of most vegetables, fruit, pulses and legumes are represented in Figure 1B and Figure 2B. There were no statistically significant differences in the portion sizes between HFI children and adults, except in green pepper (children: 45 g [15–60] vs. adults: 10 g [7.5–45]; *p* < 0.05) (Figure 1B).

In HFI patients, the median vegetable, fruit and legume servings (grams per serving) and the total fructose content of the food correlated negatively (ρ = −0.353; *p* = 0.007) (Figure S1).

## 4. Discussion

This is the first time that dietary intake and consumption patterns related to vegetables, legumes and fruit have been studied in a large cohort of children and adults with HFI. As expected, the limited consumption of fruit and vegetables in these patients resulted in markedly lower intake of fructose, sucrose, and sorbitol compared with healthy control subjects. Also, no differences were found in the frequency of consumption between HFI children and adults.

Furthermore, the FFQ, not frequently used in HFI patients, allowed us to assess the consumption of certain foods that are rarely consumed by the HFI population and barely appear in a three-day dietary survey. This method made it possible to analyze the frequency of consumption and portion sizes per food, normally affected by altered food habits in these patients, and this allowed us to provide valuable, previously unknown information about HFI patients’ eating patterns.

The accepted threshold of total fructose intake in HFI patients is not well defined, but a threshold of 40 mg/kg bw/day is accepted, with a maximum of 1–1.5 g/day. In this study, most HFI patients, including children and adults, consumed less than the maximum recommended daily amount of fructose, as in other studies with this population [24]. This is due in part to their lesser overall difficulty in adhering to dietary restrictions because of their innate aversion to sweets, fruit and vegetables [25]. In our study, only three HFI patients consumed fructose above this threshold (8.6% of HFI patients), and none of them consumed more than 60 mg/kg bw/day. Although higher fructose intakes would be expected to lead to greater liver damage in HFI patients, in a study by Di Dato et al. no significant correlation was found between fructose intake and aminotransferase levels or signs of mild liver injury in HFI patients [24], possibly because these patients had higher residual enzymatic activity and could tolerate higher amounts of fructose.

There is no consensus over whether fructose tolerance increases with age or whether there may possibly be a slight increase in fructose intake after adolescence when growth is over. Our study showed that there were no differences between children and adults in total fructose consumption, whether in the pattern of vegetable consumption or in terms of the variety, quantity or frequency of most vegetables or legumes. The FFQ in HFI patients showed a significant negative correlation between the total amount of fructose in the different foods and the frequency and portion sizes consumed. Therefore, as expected, foods with higher fructose content were consumed less and in smaller quantities.

Current HFI dietary recommendations state that HFI patients should consume a maximum of one portion of vegetables (preferably boiled because the amount of fructose is reduced by boiling) or legumes a day. Vegetables or legumes are further divided into three groups: a first group with a total fructose content below 0.5 g/100 g (these may be consumed once daily); a second group with a total fructose content of 0.5–1 g/100 g (these may be consumed at maximum 2–3 times a week, once daily); and a third group with a total fructose content higher than 1 g/100 g (these should not be consumed) [4]. In our study, HFI patients consumed more than one portion daily, with a median of 2.87 times daily in total for vegetables and fruit. Vegetables from the first group (fructose content < 0.5 g/100 g), such as mushrooms, potatoes, chard or spinach, were consumed by most HFI patients, with potatoes being the most frequently consumed vegetables (old potatoes 104 times/year [2–365] and new potatoes 104 times/year [8–730]). Other vegetables such as artichokes, arugula or lamb’s lettuce were much less frequently consumed. Some vegetables from the second group (fructose content 0.5–1 g fructose/100 g), for example other types of lettuce or asparagus, were consumed by nearly one-third of HFI patients.

In this study, we showed that in real life, HFI patients do eat some vegetables in the third group with the highest fructose content, such as broccoli, garlic and onions. Broccoli is frequently consumed because HFI patients only eat the green flower heads, which are the portion with the lowest fructose content. Furthermore, in our study, HFI patients consumed garlic and onion, because they are used in small quantities in sautés in typical Spanish cuisine.

The fructose and sucrose in vegetables and the contents of other simple sugars vary with the growing season [26], water deficits [27], storage conditions [28] or variety. For this reason, it is possible that some of these products with total amounts of fructose close to the recommended limits may change from one vegetable recommendation group to another, depending on these variables. Potatoes are a good example. Traditionally, old potatoes have been recommended over new potatoes, but our study showed that this recommendation is not followed by HFI patients. There is a wide range of sugar concentrations among different potato varieties, and the content is highly influenced by environmental conditions during cultivation, seasonal variations and storage. Sucrose, glucose and fructose are the most abundant sugars in a raw potato. The content of sucrose and glucose is high at the early stage and decreases with tuber growth. In contrast, the fructose content is low throughout the growing period and increases during storage. Further, cold stress converts sucrose into glucose and fructose, thus causing higher concentrations of fructose in cold-stored tubers (cold sweetening). Genotypes with low sucrose levels at harvest have a greater ability to control sucrose accumulation as soon as tuber development is completed, and, thus, the fructose content will be lower during transport and storage [29,30,31]. Therefore, these varieties of potatoes would be recommended for HFI patients.

In our study, although healthy control subjects consumed a greater ammount of vegetables and fruit than HFI patients, neither of the two groups reached the level of fruit and vegetable intake recommended in a Mediterranean diet [32]. Moreover, it is well known that the restriction of vegetables in the diet, along with the fact that they are usually consumed in cooked form, leads most HFI patients to consume less than the recommended amount of vitamin C [33].

In HFI patients, lentils were the most frequently consumed pulses and legumes due to their lower total fructose content. Chickpeas, despite their higher fructose content (>1 g of fructose/100 g), were consumed by 72.2% of HFI participants, but less frequently and in smaller portions than lentils. Legumes and pulses are low in fructose but higher in sucrose (in contrast to vegetables). Due to the greater sweetening power of fructose (1.8 times sweeter than sucrose [34]) HFI patients probably find pulses less sweet than vegetables with the same total amount of fructose. In general, worldwide recommendations related to beans, peas, and lentils are 1–3 servings or cups per week depending upon caloric intake [35], although new versions of the Mediterranean diet suggest an increase in their consumption up to once daily (at least one small serving per day) because of their vegetable protein content, essential amino acids, low fat content and low glycemic index [32]. In our study, there was a trend for lower weekly consumption of legumes in HFI patients compared to the healthy control subjects, and the consumption frequency was lower than current Mediterranean diet recommendations in both groups.

In recent years, in Spain, most individuals have had a protein intake largely exceeding their needs, particularly due to an increase in red meat intake [32], characteristic of the Western pattern diet. In the case of HFI patients, the limitation of vegetables, legumes and varied grains and the abuse of meat, fish, eggs and dairy products due to their lack of fructose content led to high protein consumption, mainly of animal origin, which was higher than that in the healthy control group.

Due to reduced fruit and vegetable consumption in HFI patients, their dietary fiber intake was lower than that in healthy control subjects. However, some vegetables with high amounts of fructans, FOS or inulin per 100 g, such as garlic (17.4 g), leek (7.1 g), spring onion (6.3 g), white onion (1.8 g) and artichoke (1.2 g) [36], were widely consumed by HFI patients, although only artichokes were consumed in large quantities (onions and leeks also have a high amount of fructose and sucrose). Moreover, there is some controversy over artichoke consumption, presumably due to its content of inulin, as the total amount of fructose is low. Inulin and FOS are not digested by the enzymes in the small intestine, and they are not absorbed in the gastrointestinal tract. Instead, 86–88% of the intake is recovered at the end of the small intestine, and 12–14% is hydrolyzed and fermented by bacteria in the colon, producing mainly short-chain fatty acids and gases [37]. Although a small amount of free fructose and sucrose (around 3% of total fructose) is present in some commercial inulin, a study with a small number of HFI patients showed tolerability without alterations in analytical parameters after the consumption of 6 g/m^2^/day for 2 days of commercial FOS. In this study, blood chemistry values were within normal ranges and did not change appreciably during the study period, except for two patients showing slight elevations of uric acid [38]. However, it is possible that inulin is slowly hydrolyzed into fructose in acid foods with a long shelf life and in the stomach (at pH 1.3 as much as 8% of inulin is converted into fructose in 2 h) [39]. In artichokes, the inulin content is strongly influenced by the storage temperature and preservation method. During storage (especially at higher temperatures and in storage without packing), inulin depolymerization involves a decrease in inulin content associated with an increase in fructose and sucrose [28]. Therefore, artichokes should be consumed with caution by HFI patients.

The strengths of this study include a comprehensive assessment of dietary patterns in a large nationwide cohort of HFI patients, compared to a cohort of age- and sex-paired healthy control subjects.

We acknowledge that the statistical power of this study would increase with a larger number of participants. However, we were able to enroll a fairly good number of patients and healthy control subjects, considering the low prevalence of HFI. In addition, although the same researcher instructed all the participants, the nutritional records of dietary intake might be subjective and susceptible to underreporting.

## 5. Conclusions

In this study, we assessed real-life dietary patterns in a large cohort of HFI patients, showing that HFI patients present high protein intake and that real-life eating patterns do not always coincide with the established eating recommendations. Although in most HFI patients the total amount of fructose intake was within the recommended limits (with no differences between children and adults), discrepancies from the recommendations may arise because the fructose content of vegetables and legumes is not constant and depends on many factors that are not always taken into account in the nutritional recommendations for these patients.

Further research on the fructose content of different foods, especially fruits and vegetables, and updated dietary recommendations for HFI patients are warranted to give our patients the best tools to follow the nutritional treatment of the disease.

## Figures and Tables

**Table 1:** Anthropometric and nutritional characteristics in HFI patients and healthy controls.

	Healthy Controls	HFI	*p* value *
n	28	36	
Male/female, n/n	12/16	16/20	0.899
Age, years	16.37 [2.18–61.86]	15.03 [4.55–64.25]	0.695
Weight, kg	49.72 ± 15.30	44.64 ± 16.66	0.214
BMI, kg/m^2^	20.06 ± 3.01	18.93 ± 3.18	0.155
Dietary intake			
n	28	35 **	
Kcal/day	1738.45 [1094.26–2814.64]	1813.76 [1205.41–3048.23]	0.761
Protein (g/day)	70.39 [35.21–133.83]	92.43 [65.1–165.03]	**0.001**
Fat (g/day)	78.74 [47.01–146.59]	94.1 [40.9–160.13]	0.281
Carbohydrates (g/day)	202.57 [124.9–313.61]	170.23 [68.3–348.4]	0.078
Fructose (g/day)	12.5 [1.67–23.53]	0.22 [0–1.32]	**<0.001**
Sucrose (g/day)	21.57 [8.21–52.17]	0.35 [0.02–3.75]	**<0.001**
Sorbitol (g/day)	0.55 [0.01–2.82]	0.0003 [0–0.44]	**<0.001**
Total fructose consumption (Fructose + 50% Sucrose + Sorbitol) (g/day)	22.93 [5.8–50.31]	0.41 [0.01–2.5]	**<0.001**
Plant fiber (g/day)	15.86 [9.42–59.7]	11.93 [5.48–21.4]	**<0.001**

Continuous variables are represented as mean ± standard deviation or as median [minimum–maximum] depending on data distribution. Body mass index (BMI). * Significant *p*-values are marked in bold. ** 35/36 HFI patients completed dietary intake record.

**Table 2:** Fructose, sucrose and sorbitol dietary intake in HFI patients.

Dietary Intake	HFI Patients All Together	HFI Children	HFI Adults	*p* value
n	35	21	14	
Fructose (mg/kg bw/day)	5.43 [0–32.27]	3.85 [0–32.27]	6.62 [0.71–23.57]	0.145
Sucrose (mg/kg bw/day)	7.29 [0.6–69.4]	6.74 [0.6–32.7]	8.84 [1.5–69.4]	0.678
Sorbitol (mg/kg bw/day)	0 [0–14.47]	0 [0–14.47]	0 [0–1.55]	0.495
Total fructose consumption (Fructose + 50% Sucrose + Sorbitol) (mg/kg bw/day)	9.79 [0.29–59.09]	8.66 [0.29–59.09]	11.84 [1.58–46.2]	0.325

Continuous variables are represented as median [minimum–maximum] due to data distribution. Bw, bodyweight.

**Table 3:** Vegetable intake in HFI patients.

	HFI Patients Together	HFI Children	HFI Adults	*p* value *	Total Fructose Content (Fructose + 50% of Sucrose + Sorbitol)
n	36	21	15		
**Lamb’s lettuce**	14 (38.9%)	7 (33.3%)	7 (46.7%)	0.418	0.15
**Arugula**	7 (19.4%)	1 (4.8%)	6 (40%)	**0.013**	0.15
**Spinach**	32 (88.9%)	18 (85.7%)	14 (93.3%)	0.626	0.23
**New potato**	21 (58.3%)	12 (57.1%)	9 (60%)	1.000	0.32
**Mushrooms**	31 (86.1%)	19 (90.5%)	12 (80%)	0.630	0.38
**Old potato**	29 (97.2%)	16 (76.2%)	13 (86.7%)	0.680	0.46
**Artichoke**	9 (25%)	6 (28.6%)	3 (20%)	0.705	0.48
**Escarole**	2 (5.6%)	1 (4.8%)	1 (6.7%)	1.000	0.55
**Chard**	28 (77.8%)	17 (81%)	11 (73.3%)	0.694	0.56
**Peas**	1 (2.8%)	0 (0%)	1 (6.7%)	0.417	0.61
**Watercress**	7 (19.4%)	5 (23.8%)	2 (13.3%)	0.674	0.76
**Other lettuce**	15 (41.7%)	5 (23.8%)	10 (66.7%)	**0.017**	0.76
**Radish**	1 (2.8%)	0 (0%)	1 (6.7%)	0.17	0.78
**Romaine lettuce**	11 (30.6%)	5 (23.8%)	6 (40%)	0.465	0.80
**Asparagus**	10 (27.8%)	2 (9.5%)	8 (53.3%)	**0.007**	0.85
**Cauliflower**	4 (11.1%)	0 (0%)	4 (26.7%)	**0.023**	1.01
**Iceberg lettuce**	9 (25%)	5 (23.8%)	4 (26.7%)	1.000	1.03
**Eggplant**	1 (2.8%)	0 (0%)	1 (6.7%)	0.417	1.04
**Cabbage**	2 (5.6%)	1 (4.8%)	1 (6.7%)	1.000	1.08
**Zucchini**	10 (27.8%)	6 (28.6%)	4 (26.7%)	1.000	1.09
**Celery**	9 (25%)	4 (19%)	5 (33%)	0.443	1.10
**Green pepper**	13 (36.1%)	9 (42.9%)	4 (26.7%)	0.319	1.10
**Wild asparagus**	13 (36.1%)	5 (23.8%)	8 (53.3%)	0.069	1.11
**Garlic**	25 (69.4%)	13 (61.9%)	12 (80%)	0.295	1.25
**Cucumber**	4 (11.1%)	1 (4.8%)	3 (20%)	0.287	1.35
**Green beans**	9 (25%)	6 (28.6%)	3 (20%)	0.705	1.44
**Red cabbage**	1 (2.8%)	0 (0%)	1 (6.7%)	0.417	1.56
**Leek**	11 (30.6%)	6 (28.6%)	5 (33.3%)	1.000	1.61
**Broccoli**	21 (58.3%)	12 (57.1%)	9 (60%)	0.864	1.75
**Tomato**	12 (33%)	6 (28.6%)	6 (40%)	0.473	1.86
**Turnip**	3 (8.3%)	1 (4.8%)	2 (13.3%)	0.559	1.99
**Spring onion**	2 (5.6%)	1 (4.8%)	1 (6.7%)	1.000	2.00
**Endive**	3 (8.3%)	0 (0%)	3 (20%)	0.064	2.30
**Red pepper**	3 (8.3%)	0 (0%)	3 (20%)	0.064	2.30
**Onion**	11 (30.6%)	4 (19%)	7 (46.7%)	0.141	2.55
**Carrot**	5 (13.9%)	0 (0%)	5 (33.3%)	**0.008**	3.40

* Significant *p*-values are marked in bold.

**Table 4:** Fruit, dried fruit, pulse and legume intake in HFI patients.

	HFI Patients Together	HFI Children	HFI Adults	*p* value *	Total Fructose Content (Fructose + 50% of Sucrose + Sorbitol)
n	36	21	15		
**Black olives**	19 (52.8%)	10 (47.6%)	9 (60%)	0.463	0
**Green olives**	28 (77.8%)	13 (61.9%)	15 (100%)	**0.011**	0
**Sesame seeds**	12 (33.3%)	5 (23.8%)	7 (46.7%)	0.151	0.35
**Walnuts**	10 (27.8%)	4 (19.0%)	6 (40.0%)	0.260	0.56
**Lentils**	29 (80.6%)	16 (76.2%)	13 (86.7%)	0.674	0.62
**Pumpkin seeds**	11 (30.6%)	6 (28.6%)	5 (33.3)	1.000	0.65
**Avocado**	22 (61.1%)	14 (66.7%)	8 (53.3%)	0.418	0.90
**Lime**	3 (8.3%)	3 (14.3%)	0 (0%)	0.250	0.95
**Red beans**	3 (8.3%)	1 (4.8%)	2 (13.3%)	0.559	0.99
**Black beans**	1 (2.8%)	0 (0%)	1 (6.7%)	0.417	0.99
**Sunflower seeds**	27 (75.0%)	15 (71.4%)	12 (80.0%)	0.705	1.05
**White beans**	14 (38.9%)	6 (28.6%)	8 (53.3%)	0.133	1.05
**Chickpeas**	26 (72.2%)	14 (66.7%)	12 (80.0%)	0.468	1.19
**Lemon**	21 (58.3%)	12 (57.1%)	9 (60%)	0.864	1.61
**Peanuts**	8 (22.2%)	3 (14.3%)	5 (33.3%)	0.236	2.00
**Almonds**	14 (38.9%)	6 (28.6%)	8 (53.3%)	0.133	2.10
**Cantaloupe melon**	1 (2.8%)	1 (4.8%)	0 (0%)	1.000	2.25
**Cashew nuts**	7 (19.4%)	3 (14.3%)	4 (26.7%)	0.418	3.05
**Hazelnuts**	8 (22.2%)	1 (4.8%)	7 (46.7%)	**0.005**	3.44
**Papaya**	2 (5.6%)	2 (9.5%)	0 (0%)	0.500	3.50
**Pistachios**	10 (27.8%)	5 (23.8%)	5 (33.3%)	0.709	4.05
**Chestnuts**	7 (19.4%)	4 (19.0%)	3 (20.0%)	1.000	6.15

* Significant *p*-values are marked in bold.

## Data Availability

The data presented in this study are available on request from the corresponding author due to privacy or ethical restrictions.

## Supplementary Materials

The following supporting information can be downloaded at https://www.mdpi.com/article/10.3390/nu18050771/s1, Figure S1: Correlation of the frequency of consumption and serving sizes of vegetables, fruit, dried fruit, pulses and legumes with the total fructose content of foods (fructose + 50% sucrose + sorbitol, g/100 g).
