# Supplementary material for: Dietary Patterns in a Nationwide Cohort of Patients with Hereditary Fructose Intolerance
Source: Nutrients. 2026 Feb 27;18(5):771. doi: 10.3390/nu18050771 (PMC12986441; doi:10.3390/nu18050771)
Supplement: Supplementary file 1 [file nutrients-18-00771-s001.zip › nutrients-4129635-supplementary.pdf]

MDPI

nutrients

Supplementary Figure S1

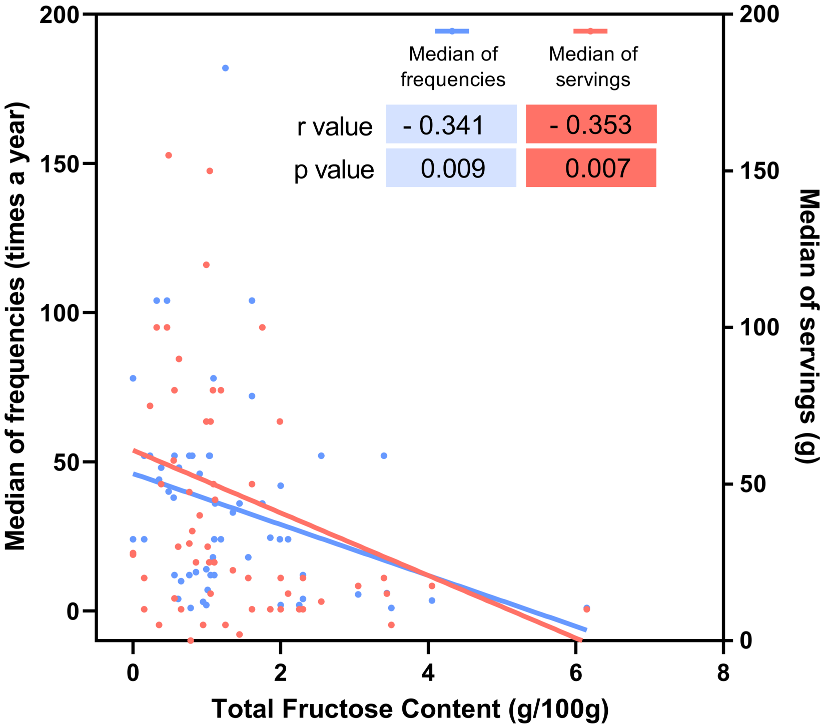

Figure S1. Correlation between the frequency of consumption and the serving size of vegetables, fruit, dried fruit, pulses and legumes with the total fructose content of foods (fructose + 50% sucrose + sorbitol, g/100 g). Blue dots represent the median frequency of consumption (times/year), and red dots represent the median serving size (g).
